# Dynamics Modeling and Experimental Investigation of Gear Mechanism with Coupled Small Clearances

**DOI:** 10.3390/e23070834

**Published:** 2021-06-29

**Authors:** Jianchao Han, Lei Liang, Huibo Zhang, Yang Zhao

**Affiliations:** 1 Department of Astronautics Engineering, Harbin Institute of Technology, Harbin 150001, China; hanjchhit@gmail.com (J.H.); yangzhao@hit.edu.cn (Y.Z.); 2 Beijing Satellite Manufacturing Factory, Beijing 100094, China; 3 School of Mechanical Engineering, Hebei University of Technology, Tianjin 300130, China; zhanghb@hebut.edu.cn

**Keywords:** small clearances, gear mechanism, dynamic modeling, experiment

## Abstract

Internal gear mechanism is widely used in micro-nano satellites due to its compact structure and high precision transmission. However, the vibration coupling caused by the small clearance coupling is more obvious and cannot be ignored under low speed, light load and zero gravity conditions. Based on the geometric relationship between radial clearance and backlash, a coupled model between dynamic backlash and radial clearance of internal meshing gear is established. Based on the conformal contact theory, the radial collision force model of the gear shaft and shaft sleeve considering the small clearances is established. Additionally, a multi-clearance gear rotor system test device is built to measure the vibration acceleration of the internal gear rotor system by an acceleration sensor and transmitted to the industrial computer through a signal collector for data processing. Through the comparison of simulation and experiment, the accuracy of the gear dynamics model is verified. The analysis results show that, compared with the traditional model, the calculation results of the gear mechanism model considering the small clearance coupling is closer to the experimental data.

## 1. Introduction

With the rapid development of micro-satellites, the transmission mechanism as the core component has become the focus of attention. The internal gear mechanism is widely used in the field of aerospace due to its compact structure and high precision. However, due to manufacturing and assembly errors, fatigue and wear, the clearance in the internal gear is inevitable. Under the conditions of low speed, light load and zero gravity, the vibration of the gear with small clearance coupling is more obvious and cannot be ignored. In addition, these clearances will lead to contact and impact between gear groups, thus affecting the control accuracy. With the development of high-speed and high-precision mechanical engineering, the motion accuracy and operational stability of mechanical systems are more demanding than ever before, and the effect of clearance on the dynamic characteristics of the system has gradually attracted the attention of scholars [1,2,3]. The previous models are too much simplified, and the object of study is relatively simple; therefore, these models are basically studied for the single clearance problem. However, there are multiple clearances in the configurations actually used in engineering. For example, there are four rotary hinge clearances in the crank-rocker mechanism; three rotary hinge clearances and one sliding clearance exist in the crank slider mechanism and the radial clearance of the bearing and the backlash of the gear are included in the gear system. Therefore, the study of the multi-clearance mechanism has great application value in mechanical structure design.

The dynamic research of gear mechanism considering multi-clearance started from Kahraman’s nonlinear model of gear system which considered both backlash and radial clearance [4,5,6,7]. Then, Byrtus et al. analyzed the influence of nonlinear factors such as clearance and time-varying mesh stiffness on the vibration characteristics of the system in detail [8]. R Farère et al. believed that it is necessary to deal with bearing clearance with the establishment of a dynamic model of gear mechanism [9]. Farshidianfar and Anooshirvan analyzed the nonlinear characteristics of multi-clearance gear mechanism [10]. Subsequently, Fernandez and others established more comprehensive multi-clearance models considering factors on this basis [11,12,13]. However, in the existing models, the radial clearance model and the backlash model of the gear are independent of each other, ignoring the influence of the radial run-out of the gear on the position of the gear tooth meshing point and the backlash, unable to describe the coupling between radial clearance and the backlash. In fact, when the radial clearance of the bearing causes the gear to run out in the gear transmission process, the actual center distance between the gear teeth changes, and the size of the meshing point and the backlash changes accordingly. In some special application backgrounds, such as aerospace agencies, low speed, light load and zero gravity will make the coupled vibration between multiple clearances more obvious and difficult to suppress. However, gear mechanisms are widely used in aerospace structures.

Therefore, a dynamic model of internal meshing gear rotor system considering multi-clearance coupling is established to further study the influence of multi-clearance coupling on the dynamic characteristics of internal meshing gear mechanism [14]. The test platform of the multi-clearance inner meshing gear rotor system was established to carry out a dynamics verification test study. Through the comparative analysis of the test data, the difference in the dynamic response between the traditional dynamic model of the multi-clearance gear mechanism and the multi-clearance model proposed in this paper is obtained.

## 2. Dynamic Model of Multi-Clearance Internal Gear Mechanism System

**Figure 1:**
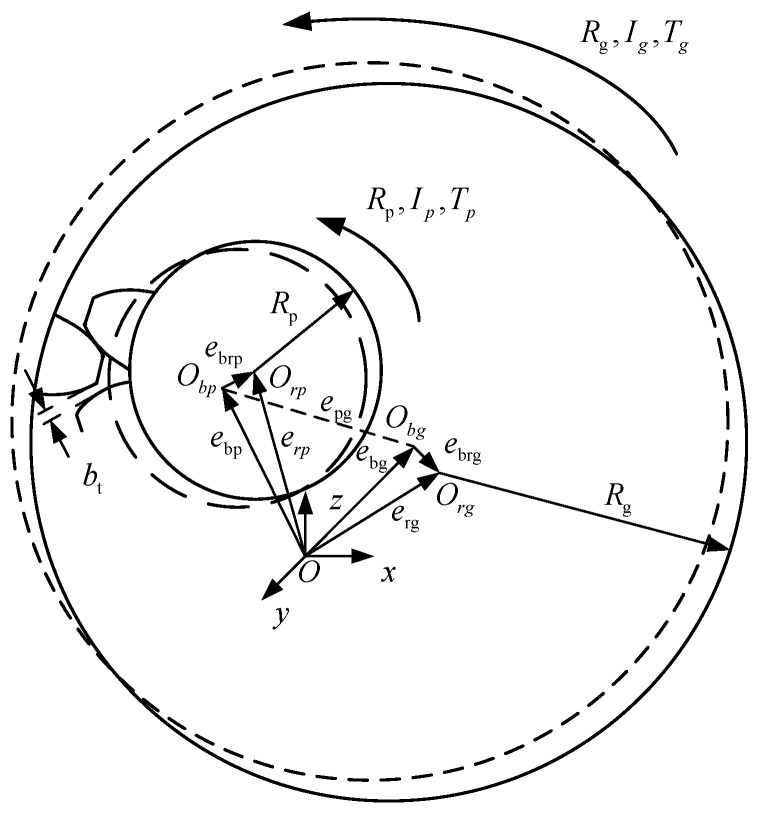
Dynamic model of gear mechanism.

An internal gear mechanism is widely used in aerospace field due to its compact structure and high precision transmission. However, in the internal meshing gear mechanism, clearances exist not only in the bearing, but also in the tooth side, which will lead to radial run out of the gear and dynamic change of the backlash, and the torsional vibration at the gear place will influence the radial vibration. Therefore, in order to reflect the phenomenon of vibration caused by coupling of multiple clearances, a nonlinear dynamic model of internal gear mechanism with multiple clearances is established, as shown in Figure 1.

Figure 1 shows the dynamic model of the gear transmission system, where is the pressure angle, *b_t_* is the dynamic backlash of gear, *e_pg_* is the center distance vector of the gear, *O_rj_* and *O_bj_* are the center of rotation and the sleeve center of the gear shafts, respectively, *e_brj_* is the clearance vector of the gears, *e_rj_* and *e_bj_* are the rotor center vector and bearing center vector of the gears, respectively, *R_j_* is the pitch radius of the gears, *I_j_* and *θ_j_* are moment of inertia and angular displacement of the gears, respectively. Here, when *j* is *p*, it represents the driving gear, when *j* is *g*, it represents the driven gear. In the dynamic modeling, the dynamic gear backlash model is first established, and then the gear torsional vibration model and the radial vibration model are given. Finally, the dynamic model of the multi-clearance gear mechanism considering the radial clearance and dynamic gear backlash is given.

### 2.1. Multi-Clearance Coupled Model

In order to establish the radial vibration model of the gear, the bearing can be simplified as a planar shaft and shaft sleeve, and the radius difference between them is the radial clearance *C_ri_*. In Figure 1, it can be represented by the radial clearance vector *e_bri_*, which is computed with the following Equation:

(1) $$e_{bri}=e_{ri}-e_{bi},$$

Further, the maximum embedding depth between the shaft to the collar is described by the clearance vector, that is, the maximum embedding amount *δ_imax_*, which can be calculated using the following relationship:

(2) $$\delta_{\mathrm{imax}}=\left\{ \begin{array}{l} \left|e_{bri}\right|-c_{ri}\;\;\left|e_{bri}\right|>c_{ri}\\ 0\;\;\;\;\;\left|e_{bri}\right|=c_{ri}\\ 0\;\;\;\;\;\left|e_{bri}\right|<c_{ri} \end{array}\right.,$$

Additionally, the gear rotor will collide with the shaft sleeve radially, causing the gear to run out radially, which will cause the gear backlash to dynamically change during the gear transmission process. The actual center distance *A_R_* of the gear can be expressed by the center distance vector *e_pg_* in Figure 1. The actual center distance *A_R_* can be calculated as:

(3) $$A_{R}=\left|e_{pg}\right|=\left|\left(e_{bp}+e_{brp})-(e_{bg}+e_{brg}\right)\right|$$

As shown in the formula, the actual center distance *A_R_* changes with the change of the clearance vector, and the change of *A_R_* will further cause the change of the size of the backlash. According to the geometric relationship of gear meshing, when the meshing position changes with the actual center distance, the dynamic backlash can be expressed as:

(4) $$b_{t}=(\frac{2\pi R_{p}^{\prime }}{Z_{p}}-s_{p}^{\prime }-s_{g}^{\prime })\cos(\alpha^{\prime })+b_{0}$$

where *R*_p_′ is the index circle radius of the driving gear, *b*_0_ is the initial backlash of gear, *Z_p_* is the number of teeth of the driving gear, *s_p_*′ is the driving gear pitch tooth thickness, *s_g_*′ is the thickness of the driven gear pitch, *α*′ is the actual meshing angle when the main and driven gear teeth mesh, as given by:

(5) $$\alpha^{\prime }=ar\cos(\frac{A_{0}}{\left|\left(e_{bp}-e_{bg})+(e_{brp}-e_{brg}\right)\right|}\cos(\alpha_{0}))$$

According to Equations (1), (3) and (4), the functional expression of the dynamic backlash *b_t_* is given as:

(6) $$b_{t}(t)=2A_{0}\cos(\alpha_{0})\cdot (inv(\alpha^{\prime })-inv(\alpha_{0}))+b_{0}$$

It can be seen from Equations (5) and (6) that in the dynamic verification test, adjusting the center distance *A_R_*, the actual meshing angle *α*′ of the gear changes and the backlash *b_t_* also changes. Additionally, the actual meshing angle *α*′ is only affected by the radial run out of the bearing, and the radial run-out amplitude can be achieved by controlling the radial radius difference between the shaft and the sleeve in the experiment.

### 2.2. Radial Collision Force Model and Gear Meshing Force Model

**Figure 2:**
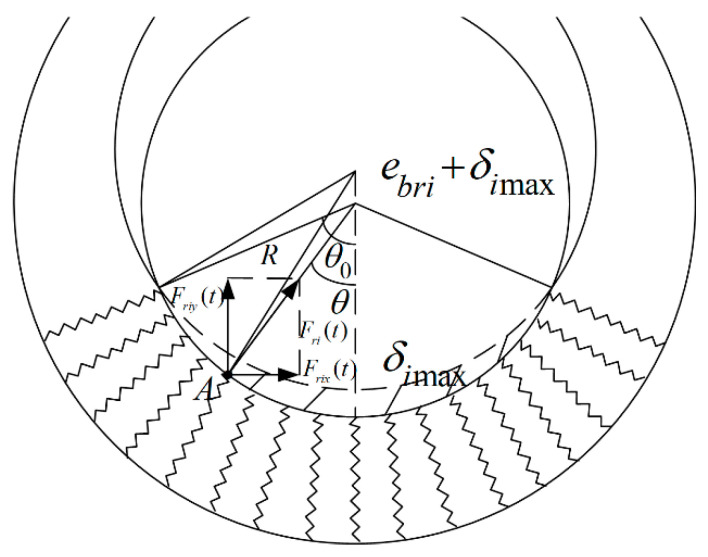
Embedded model of shaft and axle sleeve.

With reference to the Hertz model theory, the shaft is regarded as a rigid wedge, and the shaft sleeve is established with reference to the Winkler elastic foundation model, as shown in Figure 2, in which there is no interaction force between the springs.

The Hertz model is used to establish the radial impact force model of gear bearing at a contact point, the expression is:

(7) $$F_{r}(t)=K_{r}\delta_{}{}^{n}+C_{r}{\dot{\delta }}_{}$$

where *F_ri_*(*t*) is the bearing collision force *F_rp_*(*t*) and *F_rg_*(*t*) of the driving and driven gears, *K_ri_* is the contact stiffness coefficient, *n* is the force index, δ*_i_* is the amount of embedding at that point, δ*_i_* is the relative velocity in the radial direction and *C_ri_* is the damping coefficient.

According to the Equation (7), the radial force at point A can be obtained as:

(8) $$F_{ri}(t)=K_{ri}\delta_{i}^{n}+C_{ri}{\dot{\delta }}_{i}$$

In the Equation (8), the contact stiffness *K_ri_* can be expressed as:

(9) $$K_{ri}=\frac{4}{3\pi (\frac{1-v_{bi}^{2}}{\pi E_{bi}}+\frac{1-v_{ji}^{2}}{\pi E_{ji}})}{\left[\frac{R_{bi}R_{ji}}{R_{bi}-R_{ji}}\right]}^{1/2}$$

where *υ_bi_* and *E_bi_* are the Poisson’s ratio and elastic modulus of the sleeve, respectively, *υ_ji_* and *E_ji_* are the Poisson’s ratio and elastic modulus of the shaft, respectively.

The damping coefficient *C_ri_* of the radial impact force can be given by:

(10) $$C_{ri}=\frac{3K_{ri}(1-c_{ei}^{2})\delta_{i}^{m}}{4\overset{\cdot }{\delta_{i}^{\left(-\right)}}}$$

where *c_ei_* is the coefficient of restitution, which can be used to describe the energy loss during a collision, ${\dot{\delta }}_{i}^{\left(-\right)}$ is the relative speed at the critical collision.

The radial force in *y* direction of *j* at any contact point between the shaft and the sleeve can be written as:

(11) $$F_{riy}(t)=(K_{ri}\delta_{ij}^{n}+C_{ri}{\dot{\delta }}_{ij})\cos\theta ,$$

Insertion amount of any contact point between shaft and sleeve can be represented as follows:

(12) $$\begin{array}{cc} \delta ij= & R-[{\left(R+e_{bri}\right)}^{2}+{\left(e_{bri}+\delta_{imax}\right)}^{2}\\ & -2(R+e_{bri})(e_{bri}+\delta_{imax})\cos\theta {]}^{1/2} \end{array}$$

where *R* is the radius of the shaft.

The angle between the contact boundary point of shaft and sleeve and the vertical direction of shaft axis can be expressed as:

(13) $$\;\theta_{0}=\pi -arccos\frac{R^{2}+{\left(e_{bri}+\delta_{imax}\right)}^{2}-{\left(R+e_{bri}\right)}^{2}}{2R\left(e_{bri}+\delta_{imax}\right)},$$

The resultant force of radial force can be calculated using:

(14) $$F_{ri}\left(t\right)=2\int \begin{array}{l} \theta_{0}\\ 0 \end{array}\left(K_{ri}\delta_{ij}{}^{n}+C_{ri}{\dot{\delta }}_{ij}\right)\cos\theta d\theta$$

The dynamic meshing force *Ft*(*t*) between the driving and driven gears can be written as follows:

(15) $$F_{t}(t)=K_{t}(t)\cdot f_{g}(t)+C_{t}(t)\cdot {\dot{g}}_{t}(t)$$

where the right side of the equation is the elastic meshing force and damping force of the gear, respectively. In elastic meshing force, *K_t_*(*t*) is the time-varying meshing stiffness, which is a periodic function used to express the influence of the change of the number of coincident teeth on the meshing stiffness when the gear teeth are meshed. The time-varying meshing stiffness *K_t_*(*t*) caused by Kahraman [5] can be computed by:

(16) $$K_{t}(t)=k_{m}+k_{\alpha }\cos(\omega_{m}t+\varphi )$$

where *k_m_* is the average meshing stiffness, *k_a_* is the stiffness amplitude, *φ* is the initial phase and *ω_m_* is the meshing frequency, which is a function related to the number of teeth and speed.

The main parameter of the meshing damping force in Equation (15) is the nonlinear damping coefficient *C_t_*(*t*), which is used to describe the energy loss. The nonlinear damping coefficient *C_t_*(*t*) can be obtained from:

(17) $$C_{t}(t)=\{\begin{array}{c} C_{m}\\ C_{m}\cdot (1-\beta^{2}(3-2\beta ))\\ 0 \end{array}\begin{array}{l} f_{g}(t)\geq d\\ 0\leq f_{g}(t)<d\\ f_{g}(t)<0 \end{array}$$

where *C_m_* is the maximum damping coefficient, *d* is the maximum embedding amount and *β* is the embedding ratio, which can be expressed as: $\beta =f_{g}(t)/d$.

In Figure 1, the master and driven gears move in three directions in the coordinate system, namely translation along the *x* direction, translation along the *y* direction and rotation around the *z* axis. The components of the clearance vector in the x and y directions can be expressed as ***e****_brpx_*, ***e****_brpy_*, ***e****_brgx_* and ***e****_brgy_*. When considering the radial movement of the gear, the relative meshing displacement can be written as:

(18) $$\begin{array}{l} g_{t}(t)=R_{p}^{\prime }\cdot \theta_{rp}-R_{g}^{\prime }\cdot \theta_{rg}+(e_{brpx}-e_{brgx})\sin\alpha^{\prime }+\\ (e_{brpy}-e_{brgy})\cos\alpha^{\prime } \end{array}$$

Through the clearance function of Kahraman [15], the relative meshing relationship between the driving and driven gears can be obtained as:

(19) $$f_{g}(t)\{\begin{array}{c} g_{t}(t)\\ 0\\ g_{t}(t)+b_{t}(t) \end{array}\begin{array}{l} g_{t}(t)\geq 0\\ -b_{t}(t)<g_{t}(t)<0\\ g_{t}(t)<-b_{t}(t) \end{array}$$

when the relative displacement is in the state of *g_t_* ≥ 0, the main and driven gears mesh normally, when the relative displacement is in the state of −*b_t_* < *g_t_* < 0, the main and driven gears are in a state of disengagement, when the relative displacement is in the state of *g_t_* ≤ −*b_t_*, tooth back collision occurs between the driving and driven gears.

### 2.3. Gear System Dynamic Mode

According to the characteristics of the multi-clearance gear mechanism, a dynamic model of the gear mechanism system considering multi-clearance coupling can be established, which can be calculated as follows:

(20) $$\{\begin{array}{c} I_{p}\cdot {\ddot{\theta }}_{\mathrm{rp}}+F_{t}(t)\cdot {R^{\prime }}_{p}=T_{p}\;\;\;\;\;\;\;\\ m_{p}\cdot {\ddot{\delta }}_{px}-F_{rpx}(t)+F_{tpx}(t)=0\;\;\;\;\\ m_{p}\cdot {\ddot{\delta }}_{py}-F_{\mathrm{rp}y}(t)+F_{tpy}(t)=0\;\;\\ F_{t}(t)\cdot R_{g}^{\prime }-I_{g}\cdot {\ddot{\theta }}_{\mathrm{rg}}=T_{g}\;\;\;\;\;\;\;\;\;\;\;\\ m_{g}\cdot {\ddot{\delta }}_{gx}-F_{rgx}(t)+F_{tgx}(t)=0\;\;\;\;\;\;\;\;\\ m_{g}\cdot {\ddot{\delta }}_{gy}-F_{\mathrm{rg}y}(t)+F_{tgy}(t)=0\;\; \end{array}$$

where ${\ddot{\theta }}_{\mathrm{rp}}$ and *m_p_* are the angular acceleration and mass of the driving gear, respectively, ${\ddot{\theta }}_{\mathrm{rg}}$ and mg are the angular acceleration and mass of the driven gear, respectively, *T_p_* is the driving torque and *T_g_* is the load torque, which is not added in the dynamic verification, only the dynamic characteristics of the driven gear under free motion are considered. *g* is the acceleration of gravity (it can also represent the acceleration under microgravity conditions). When the organization is placed horizontally, *g* = 0, where ${\ddot{\delta }}_{gx}$ ${\ddot{\delta }}_{gx}$, ${\ddot{\delta }}_{px}$ ${\ddot{\delta }}_{gy}$, ${\ddot{\delta }}_{px}$ and ${\ddot{\delta }}_{py}$ are the component of the translational acceleration of the driving gear and the driven gear on the x and y axes in the global coordinate system, respectively. *F_rpx_*(*t*), *F_rpy_*(*t*), *F_rgx_*(*t*) and *F_rgy_*(*t*) are the component of the bearing collision force, respectively, *F_tpx_*(*t*), *F_tpy_*(*t*), *F_tgx_*(*t*) and *F_tgy_*(*t*) are the component of gear meshing force, respectively.

## 3. Analysis of Vibration Characteristics of Gear Rotor System

In this section, through the combination of simulation and experimental research, the non-coupled model, the coupled model and the experimental data are compared to verify the correctness of the multi-clearance coupled theoretical model. Study on vibration characteristics of gear rotor system under different working conditions was done by changing rotational speed and clearance size.

**Figure 3:**
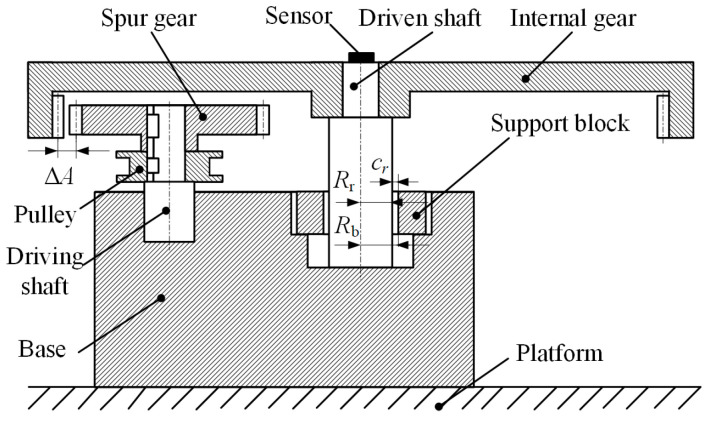
The structure of multi-clearance coupled experimental setup.

The specific structure of the test device, as shown in Figure 3, mainly includes driving gear, driving gear shaft, driven internal gear, internal gear frame, support block, base, pulley, etc. Among them, the driving gear and the driven internal gear are all involute gear, the support block is placed in the groove of the base, the internal gear and the internal gear frame are fixed together and the center hole of the internal gear frame is assembly with the cylinder on the support block. In the test, the center distance and shaft hole clearance of the main and driven gears can be adjusted by changing the support blocks with different sizes and specifications, so as to change the backlash and radial size. In the test, the driven gear was not loaded, so as to be able to simulate low speed, light load and zero gravity, and then to study the phenomenon of radial collision and tooth side collision in a free state. In the aerospace structure, the execution end (except the control moment gyro) generally has a speed below 1 rpm. Considering the deceleration capacity of the reducer, the input speed generally does not exceed 180 rpm. The horizontal arrangement of the gear shaft in most gear tests is not adopted in the test device, the arrangement of the gear perpendicular to the horizontal plane is adopted in order to equivalent the space microgravity environment, so as to ensure that the rotation plane of the gear is not affected by gravity, and the radial collision is freer. 

**Figure 4:**
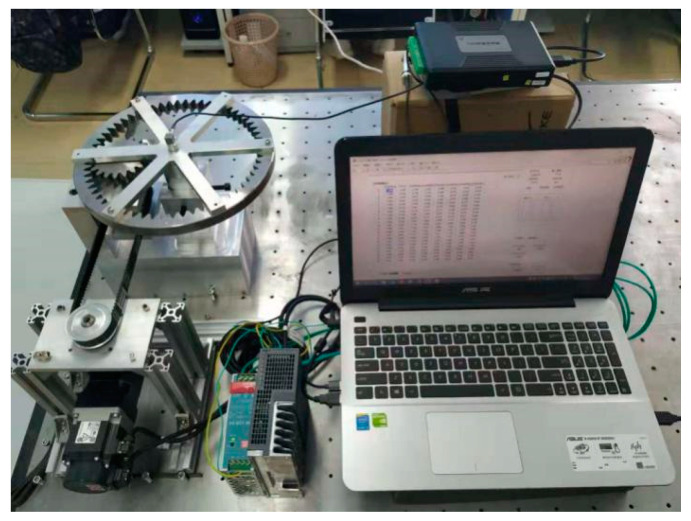
The multi-clearance coupled experimental setup.

After the schematic design of the test device is completed, the actual design, processing and assembly problems should be considered. In addition, there are several factors to consider. First of all, it is the selection of gears, because the adjustment of gear backlash is realized by changing the actual center distance of the two gears. According to Equation (6), the proportional relationship between the increase of center distance that can be written as $\Delta A\Delta A=A^{`}-A$ and gear backlash is about 1:1. Therefore, in order to ensure that the backlash can be adjusted in a larger range, a gear with a larger modulus must be selected, so that the tooth height is longer, and the center distance will not be too large to be unable to mesh. Finally, a gear with a gear module of 4 is selected. Second, the experimental device must be able to achieve adjustable speed to study the influence of different speeds on the dynamic response of the system, so a DC servo motor is selected, and the adjustable range of speed is 0~300 rpm. Finally, the power is transmitted through the synchronous belt, and the motor is installed separately from the base to avoid the influence of the vibration of the motor itself on the experimental data collection. Considering the gear inertia and the size of the entire device, a spur gear with 20 teeth and an internal gear with 60 teeth are selected. Through repeated design and verification, a multi-clearance gear rotor system test device was finally developed. The actual product is shown in Figure 4.

On this basis, the multi-gear rotor system test platform was built, including the multi-gear rotor system test device, motor driver, three-axis acceleration sensor, signal collector, host computer, etc. Among them, the motor driver is used to control the motor speed to realize the adjustable speed of the driving gear. The three-axis acceleration sensor is used to measure the vibration of the system, which is installed on the shaft of the driven gear, and the acceleration along the center line is measured emphatically. In this direction, not only the vibration frequency of the contact and collision between the shaft and the gear can be observed, but also the component of the gear meshing vibration in this direction can be observed. The acceleration sensor transmits the acceleration signal collected from the driven wheel to the upper computer through the signal collector, and the upper computer is used to further analyze the spectral characteristics. The characteristics of the test system are that it can easily adjust the size of the bearing radial clearance and backlash, and can control the driving speed, and then study the vibration characteristics of the gear rotor system under multi-clearance coupling. The main parameters of the equipment used in the test platform are shown in Table 1.

The dynamic verification test is carried out on the test platform of multi-clearance gear rotor system, and the difference of dynamic characteristics between the multi-clearance coupled model proposed in this paper and the traditional multi-clearance model is compared and analyzed based on the test data. Among them, the comparison model based on Kahraman’s multi-clearance gear system dynamics model [4,5,6,7], in the radial clearance, considering the nonlinear contact stiffness and nonlinear damping coefficient, and in the gear meshing, considering the time-varying meshing stiffness and damping characteristics. However, it is considered that the backlash is a constant, which is mainly related to the machining and assembly, precision without considering the dynamic change of backlash caused by the motion deviation of the radial backlash. That is to say, the dynamic equation of the system is basically the same as that in Equation (7), but the backlash is a constant. The multi-clearance coupled model and multi-clearance non-coupled model are used to compare with the test data in the test verification process. The specific structural parameters of the gear are shown in Table 2.

According to the above parameters, the corresponding numerical calculation and test are carried out, and the numerical calculation results are compared with the test results. In the test, the acceleration of the active gear is mainly controlled by the motor and has a negligible effect on the experimental results, so the vibration acceleration of the driven gear shaft is mainly measured. Therefore, the calculated data are also obtained in the numerical calculation, and then the frequency spectrum analysis is performed to facilitate comparison with the test data.

### 3.1. Study on Dynamic Characteristics of Gear Considering Multi-Clearance Coupling at Different Rotation Speeds

**Figure 5:**
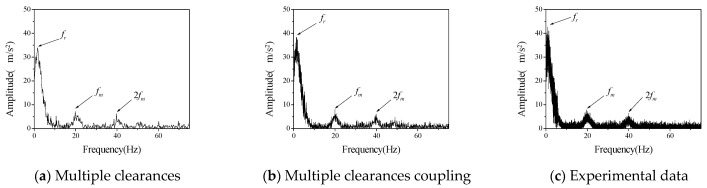
The vibration acceleration spectrum of driven gear (*c_r_* = *b*_0_ = 100 μm, *ω_p_* = 60 rpm).

**Figure 6:**
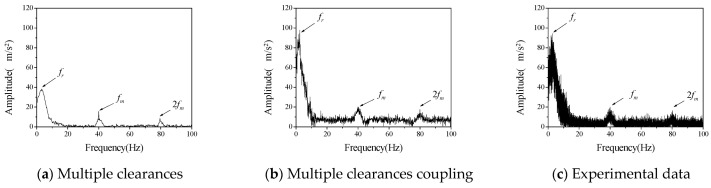
The vibration acceleration spectrum of driven gear (*c_r_* = *b*_0_ = 100 μm, *ω_p_* = 120 rpm).

**Figure 7:**
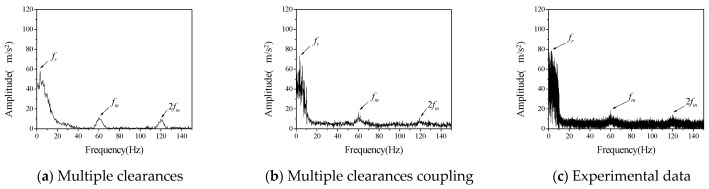
The vibration acceleration spectrum of driven gear (*c_r_* = *b*_0_ = 100 μm, *ω_p_* = 180 rpm).

First, we studied the influence of different speeds on the vibration characteristics of the system. The bearing radial clearance *c_r_* and the initial backlash *b*_0_ were considered to be 100 μm, the driving speed *ω_p_* of the driving gear was 60 rpm, 120 rpm and 180 rpm, respectively. The comparison between numerical calculation data and experimental data is shown in Figure 5, Figure 6 and Figure 7.

Acceleration data obtained in the experiment are transformed into frequency spectrum by Fourier transform. Vibration spectrums of driven gears at different rotational speeds are shown in Figure 5, Figure 6 and Figure 7, respectively. Therefore, the ordinate uses the acceleration amplitude to represent the vibration characteristics of the system. According to the rotational speed and gear structure parameters, the corresponding frequencies of the three vibration peaks are, respectively; the radial vibration frequency *f_r_* between the inner gear support frame and the support block, which is mainly caused by the continuous contact between the inner hole of the inner gear support frame and the cylindrical part of the support block, gear meshing frequency *f_m_* is caused by meshing in and out of gear teeth, the gear meshing frequency is 2 *f_m_*, which is mainly caused by the existence of nonlinear factors such as backlash and radial clearance. In the numerical calculation, if the above three frequencies can be reflected in the frequency spectrum of multi-clearance coupled model and non-coupled model, which can tell the two models are correct in calculating the basic vibration law of gear. Judging from the changing trend of amplitude with speed in the above three figures, when the speed is 120 rpm in the test data, the amplitude of radial vibration and the meshing vibration and its multiplier are significantly higher than those at the other two speeds, but the frequency characteristics are still consistent with the other two sets of data, so the influence of test errors can be excluded. This phenomenon is the vibration caused by the coupling of radial clearance and the backlash, which makes the amplitude suddenly increase. From the frequency spectrum of the coupled model and the uncoupled model, it can be seen that the amplitude of the coupled model also appears a similar phenomenon, when the speed is 120 rpm, the amplitude is larger but does not appear in the uncoupled model. The amplitude of the uncoupled model increases with the increase of rotational speed, and this trend is also quite different from the experimental data.

It can be seen from the above qualitative analysis that due to the simultaneous interaction of radial clearance and backlash in the test, the system vibration caused by the coupling of radial clearance and the backlash, leads to a certain degree of small fluctuations in the frequency spectrum. From the perspective of experiment, it is shown that there is indeed a coupling phenomenon between radial clearance and backlash in the gear mechanism, which will adversely affect the vibration characteristics of the system. The multi-clearance coupled model proposed in this paper considers the coupling relationship between radial clearance and dynamic lateral clearance, which can reflect the nonlinear phenomenon caused by multi-clearance coupling in the test. Compared with the multi-clearance uncoupled model, it is more practical.

Furthermore, the difference between the numerical calculation and the experimental data is analyzed and compared mainly in terms of frequency and amplitude, as shown in Table 3 and Table 4. 

It can be seen from Table 3 and Table 4 that the error between the coupled model and the uncoupled model in the calculation of radial vibration frequency and engagement vibration frequency is smaller than that of the test data, but there is a big difference between the uncoupled model and the test in the calculation of the amplitude, especially when the rotation speed is 120 rpm, the calculation error is very large, indicating that the uncoupled model cannot accurately reflect the coupling vibration that may occur in the multi-clearance gear mechanism. The amplitude error of the coupled model is relatively small, and it can reflect the vibration caused by the coupling of radial clearance and the backlash at 120 rpm, which indicates that the coupled model can reflect the vibration characteristics of multi-clearance gear mechanism more accurately due to the consideration of the coupling relationship between radial clearance and dynamic lateral clearance and is more in line with the reality than the traditional multi-clearance model.

### 3.2. Study on Gear Dynamic Characteristics Considering Multi-Clearance Coupling under Different Clearance Sizes

**Figure 8:**
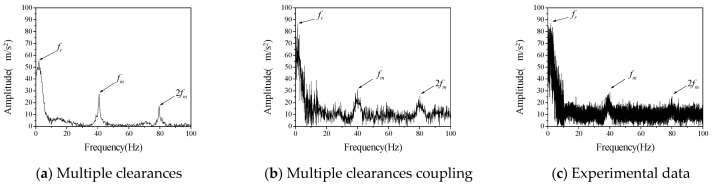
The vibration acceleration spectrum of driven gear (*c*_r_ = 200 μm, *b*_0_ = 100 μm, *ω*_p_ = 120 rpm).

**Figure 9:**
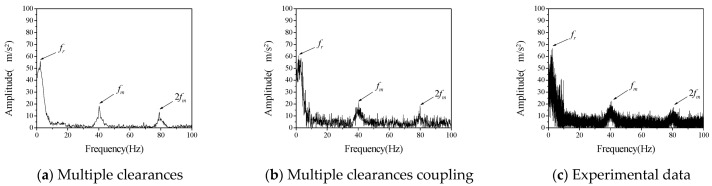
The vibration acceleration spectrum of driven gear (*c*_r_ = 100 μm, *b*_0_ = 200 μm, *ω*_p_ = 120 rpm).

**Figure 10:**
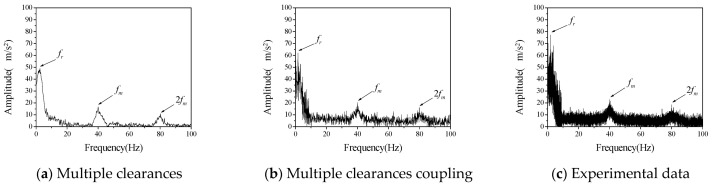
The vibration acceleration spectrum of driven gear (*c*_r_ = 200 μm, *b*_0_ = 200 μm, *ω*_p_ = 120 rpm).

The driving speed was set as 120 rpm to further verify the vibration characteristics of a gear rotor system with different radial clearance and backlash. By changing the size and position of the cylindrical part of the supporting block at the driven gear, the experimental data of three different clearance combinations are obtained, namely *c*_r_ = 200 μm, *b*_0_ = 100 μm; *c_r_* = 100 μm, *b*_0_ = 200 μm; *c_r_* = 200 μm, *b*_0_ = 200 μm. The numerical calculation values and test results of its vibration frequency spectrum are shown in Figure 8, Figure 9 and Figure 10, respectively.

Figure 8, Figure 9 and Figure 10 shows the vibration acceleration spectrum of driving gear with different clearances, which reflects the vibration characteristics of the system with different combinations of radial clearance and backlash. It can be seen from the test data of different clearance combinations that the amplitude of gear is obviously higher than that of the other two clearance combinations when the radial clearance *c_r_* = 200 μm and the initial backlash *b*_0_ = 100 μm, which indicates that the coupling vibration of the system is caused by the multi-clearance collision under this clearance combination. However, the amplitude of the uncoupled model varies from the experiment to some extent. The uncoupled model reflects that the vibration amplitude of the system is the largest when *c_r_* = 200 μm and *b*_0_ = 200 μm, there is no obvious coupling vibration in the system. It can be seen that the multi-clearance coupled model is in good agreement with the experimental data in the calculation of the influence of the clearance change on the vibration characteristics of the system, which can reflect the coupling vibration phenomenon of the system when *c_r_* = 200 μm and *b*_0_ = 100 μm.

Furthermore, the difference between numerical calculation and experimental data is analyzed quantitatively, mainly from the frequency and amplitude, as shown in Table 5 and Table 6. It can be seen that the error between the calculation of radial vibration frequency and meshing vibration frequency of multi-clearance coupled model and non-coupled model and the test data is small, but the error between the calculation of amplitude of non-coupled model and the test is large, especially when the clearance size is *c_r_* = 200 μm and *b*_0_ = 100 μm, the error is large, indicating that the non-coupled model cannot accurately reflect the coupling vibration phenomenon of multi-clearance gear mechanism. The amplitude error of the coupled model is relatively small and can reflect the vibration phenomenon caused by the coupling of radial clearance and the backlash when *c_r_* = 200 μm and *b*_0_ = 100 μm.

In summary, in the test of gear mechanism considering multiple clearances, a coupling vibration phenomenon was observed and is caused by the interaction of the radial bearing clearance and the dynamic backlash on the gear. Through the analysis of test data, it is proved that the coupled model proposed in this paper can more accurately reflect this coupling vibration phenomenon. Additionally, it is also more accurate in the calculation of frequency and amplitude. However, the coupling relationship between radial clearance and backlash was not considered in the non-coupled model, and the dynamic change of backlash was ignored, resulting in a large clearance with the test results.

Through the experimental analysis, it is found that there is still a certain error between the theoretical calculation and the experimental data. The analysis shows that the error source is mainly caused by the following reasons:

(1) In the numerical calculation, the selection of contact stiffness and damping is calculated by the empirical formula, and there is a certain error with the actual device, which directly leads to the deviation between the calculation of collision force and the actual collision force that ultimately leads to the inaccurate calculation of vibration amplitude.

(2) Due to the limitation of the machining accuracy of the driven wheel shaft and sleeve, the surface roughness and the overall morphology are different from the theoretical model, which is one of the reasons for the large clearance between the vibration amplitude in the test and the theoretical calculation. Thus, machining precision of shaft can be further improved to reduce test error.

## 4. Conclusions

In this paper, dynamical model of gear mechanism with multiple clearances coupling is investigated by use of experimental test and theoretical modeling. The following conclusions can be summarized as:

(1) A new dynamic model of the gear-rotor system considering small clearance coupling is established. This model considers nonlinear problems such as bearing radial clearance, dynamic backlash and time-varying meshing stiffness, which can more comprehensively reflect the influence of multi-clearance coupling effect on system dynamic characteristics.

(2) Through the dynamic test of multi-clearance gear mechanism, it is found that there exists a coupling vibration phenomenon in multi-clearance gear mechanism with bearing radial clearance and backlash, which will adversely affect the vibration characteristics of the system.

(3) By comparing the theoretical data with the experimental data, it is found that the multi-clearance coupled model proposed in this paper can well reflect the coupling vibration characteristics of the system. Compared with the traditional multi-clearance model, the calculation of vibration frequency and amplitude is in better agreement with the experimental data.

Influence of multi-clearance coupling on multi-stage gears transmission will be further studied by experiment and simulation analysis.

## Figures and Tables

**Table 1:** The parameters of experiment test.

Equipment Name	Model	The Main Parameters	Value
DC servo Motor	MR-JE-20A	Power (W)	200
		Voltage (V)	220
Acceleration sensor	356A02	Sensitivity (mv/(m/s^2^))	1.02
		Range (m/s^2^)	±4900
Signal collector	USB3200	Sampling frequency (kHz)	100

**Table 2:** Structure parameter of gear-rotor system.

Parameter	Value	Parameter	Value
Number of driving gear teeth	20	Quality of driving wheel kg	0.235
Number of driven gear teeth	60	Quality of driven wheel kg	0.800
Modulus	4	rotational inertia of the driving wheel kg·m^2^	0.0189
Pressure angle°	20	Rotational inertia of the driven wheel kg·m^2^	0.0612

**Table 3:** The frequency error between calculation and measurement of driven gear under different rotational speed.

Rotating Speed (rpm)	Test Results (Hz)	Coupled Model		Uncoupled Model	
		Frequency (Hz)	Error (%)	Frequency (Hz)	Error (%)
60	Radial vibration: 1.642	1.327	19.18	1.289	21.49
60	1 times frequency of meshing vibration: 19.885	19.959	0.37	19.767	0.59
60	2 times frequency of meshing vibration: 39.991	39.926	0.14	39.765	0.56
120	Radial vibration: 2.856	2.471	15.58	3.253	13.90
120	1 times frequency of meshing vibration: 39.991	39.935	0.14	40.341	0.88
120	2 times frequency of meshing vibration: 80.172	79.293	1.09	79.532	0.80
180	Radial vibration: 3.033	3.278	8.08	3.344	10.25
180	1 times frequency of meshing vibration: 60.112	60.221	0.18	59.782	0.549
180	2 times frequency of meshing vibration: 120.015	119.845	0.14	120.311	0.25

**Table 4:** The frequency error between calculation and measurement of driven gear under different rotational speed.

Rotating Speed (rpm)	Test Results (m/s^2^)	Coupled Model		Uncoupled Model	
		Amplitude (m/s^2^)	Error (%)	Amplitude (m/s^2^)	Error (%)
60	Radial vibration: 42.673	38.154	10.59	33.921	20.51
60	1 times frequency of meshing vibration: 7.961	8.116	6.94	7.209	9.45
60	2 times frequency of meshing vibration: 6.404	6.268	2.12	6.236	2.62
120	Radial vibration: 94.282	97.852	3.79	38.327	59.35
120	1 times frequency of meshing vibration: 20.152	20.307	0.77	15.831	21.44
120	2 times frequency of meshing vibration: 16.622	17.132	8.48	8.639	48.03
180	Radial vibration: 78.746	73.499	6.67	58.276	25.99
180	1 times frequency of meshing vibration: 18.317	17.137	6.44	11.348	38.05
180	2 times frequency of meshing vibration: 14.378	11.382	20.84	9.819	31.71

**Table 5:** The amplitude error between calculation and measurement of driven gear under different clearance size.

Clearance Size		The Test Results (Hz)	Coupled Model		Non-Coupled Model	
Radial Direction (μm)	Tooth Side (μm)		Frequency (Hz)	Error (%)	Frequency (Hz)	Error (%)
200	100	The radial vibration: 1.879	1.591	15.33	2.212	17.72
200	100	Meshing 1 times frequency: 40.109	40.122	0.03	40.753	1.61
200	100	Meshing 2 times frequency: 80.111	80.213	0.13	79.533	0.72
100	200	diametral vibration: 2.463	1.732	29.67	2.250	8.65
100	200	Meshing 1 times frequency: 40.895	40.330	1.38	40.253	1.57
100	200	Meshing 2 times frequency: 80.719	79.926	0.98	79.114	1.99
200	200	The radial vibration: 1.920	1.700	11.46	2.512	30.83
200	200	Meshing 2 times frequency: 40.094	40.167	0.18	40.122	0.07
200	200	Meshing 2 times frequency: 80.124	79.916	0.26	80.250	0.15

**Table 6:** The amplitude error between calculation and measurement of driven gear under different clearance size.

Clearance Size		The Test Results (m/s^2^)	Coupled model		Non-Coupled Model	
Radial Direction (μm)	Tooth Side (μm)		Frequency (m/s^2^)	Error (%)	Amplitude (m/s^2^)	Error (%)
200	100	The radial vibration: 86.923	86.488	0.50	55.173	36.53
200	100	Meshing 1 times frequency: 29.065	31.228	7.44	27.489	5.42
200	100	Meshing 2 times frequency: 25.347	25.899	2.18	16.976	33.03
100	200	The radial vibration: 66.735	60.554	9.26	51.248	23.21
100	200	Meshing 1 times frequency: 22.395	21.274	5.00	18.243	18.54
100	200	Meshing 2 times frequency: 17.121	18.506	8.09	13.301	22.31
200	200	The radial vibration: 77.265	62.095	19.63	48.947	36.65
200	200	Meshing 1 times frequency: 22.798	20.289	11.01	16.719	26.67
200	200	Meshing 2 times frequency: 18.546	16.245	12.41	10.845	41.52

## Data Availability

Not applicable.
